# Acceptance of hospital diets and nutritional status among inpatients with cancer

**DOI:** 10.1590/S1679-45082013000100008

**Published:** 2013

**Authors:** Daiane Ferreira, Tessa Gomes Guimarães, Aline Marcadenti

**Affiliations:** 1 Hospital Nossa Senhora da Conceição, Porto Alegre, RS, Brazil; 2 Universidade Federal do Rio Grande do Sul, Porto Alegre, RS, Brazil

**Keywords:** Nutritional assessment, Diet, Eating, Nutritional status, Neoplasms

## Abstract

**Objective:**

To verify acceptance of hospital diets as to the nutritional status among patients admitted to the Oncology/Hematology Unit of a tertiary care hospital.

**Methods:**

A cross-sectional study conducted among 100 patients, aged ≥18 years, of both genders. Body mass index and subjective global nutritional evaluation by patients were used to detect the nutritional status. The rest-ingestion index was used to evaluate diet acceptance, and the reasons for non-acceptance were identified by means of a questionnaire. Data were expressed in means and standard deviation, or medians and percentages. Comparisons were made using the Student's *t* test, Wilcoxon Mann-Whitney test, and Pearson's χ^2^ test.

**Results:**

A total of 59% of patients were males, and mean age was 51.6±13.5 years. According to the global subjective nutritional evaluation done by the patients themselves, 33% of the participants were considered malnourished and the body mass index detected 6.3% of malnutrition. The main symptoms reported were lack of appetite, xerostomia (dry mouth), constipation, dysgeusia, odor-related nausea, and early satiety. The rest-ingestion index was approximately 37% and significantly greater among the malnourished relative to the well-nourished (58.8 *versus* 46.4%; p=0.04). The primary reasons reported for non-acceptance of the diet offered were lack of flavor, monotonous preparations, large quantities offered, lack of appetite, and inappropriate temperature of the meal.

**Conclusion:**

A high the rest-ingestion index was seen among the patients with cancer, especially those who were malnourished according to the global nutritional evaluation produced by the patient.

## RESUMO

**Objetivo:**

Verificar a aceitação de dietas hospitalares, em relação ao estado nutricional, entre pacientes com câncer admitidos na Unidade de Oncologia/Hematologia de um hospital terciário.

**Métodos:**

Estudo transversal conduzido entre 100 pacientes, com idade ≥18 anos de ambos os gêneros. Índice de massa corporal e avaliação nutricional subjetiva global produzida pelo próprio paciente foram utilizados para detecção do estado nutricional. Índice de resto-ingestão foi utilizado para avaliar a aceitação da dieta, e os motivos para não aceitação foram verificados por meio de questionário. Os dados foram expressos em médias e desvio padrão, ou medianas e percentuais. As comparações foram realizadas por meio de testes *t* de Student, Wilcoxon Mann-Whitney e do χ^2^ de Pearson.

**Resultados:**

No total, 59% dos pacientes eram do gênero masculino e a idade média foi de 51,6±13,5 anos. De acordo com a avaliação nutricional subjetiva global produzida pelo próprio paciente, 33% dos participantes foram considerados desnutridos e o índice de massa corporal detectou 6,3% de desnutrição. Os principais sintomas relatados foram: inapetência, xerostomia, constipação, disgeusia, náuseas relacionadas aos cheiros e saciedade precoce. O índice de restoingestão foi de aproximadamente 37% e significativamente maior entre os desnutridos comparativamente aos bem nutridos (58,8 x 46,4%; p=0,04). Quanto aos motivos relatados para a não aceitação da dieta oferecida, destacaram-se falta de sabor, monotonia das preparações, grandes quantidade oferecidas, falta de apetite e temperatura inadequada da refeição.

**Conclusão:**

Observou-se um elevado índice de resto-ingestão entre os pacientes com câncer, principalmente entre os desnutridos, pela avaliação nutricional subjetiva global produzida pelo próprio paciente.

## INTRODUCTION

Malnutrition is a frequent complication among patients with cancer, associated with a decreased response to specific antineoplasic treatments, reduced quality of life, increased risks of infection, length of stay at hospital, morbidity and mortality^(1)^. It is common to observe malnutrition among hospitalized individuals, which is three times more frequent in patients with cancer when compared to those with no such diagnosis, suggesting that the disease itself hinders the nutritional status during admission at hospital^(2)^.

Various factors are involved in the genesis of malnutrition among patients with cancer, especially the effects of the treatment chosen for the disease. Frequently, individuals undergoing chemotherapy and/or radiation therapy have gastrointestinal complaints, such as nausea, vomiting, change in taste, mucositis, constipation and/or diarrhea, which can diminish acceptance of the diet and consequently, impairing the nutritional status^(1)^. In this way, an investigation of the impacts of these symptoms on food ingestion becomes indispensable for planning an early and more effective nutritional intervention in hospitalized individuals and those with the disease.

Inadequate food ingestion also contributes towards malnutrition in the hospital environment^(3)^. Reduced food intake is often reported among hospitalized patients, a fact that may be related to the disease, to changes in eating habits, and to dissatisfaction with the preparations offered. Evaluation of food consumption among patients with cancer should be routine, since eating is an important part of the treatment, not only for its nutritional aspects, but also due to its symbolic and subjective dimensions^(4,5)^.

Detection of nutritional risk by means of appropriate tools^(6,7)^ is fundamental in identifying individuals at risk for malnutrition, and early nutritional intervention would avoid the occurrence and worsening of this condition. These methods are used for screening; for a more comprehensive analysis of the nutritional status, we recommend the association of different evaluation methods (objective and subjective)^(8)^. Patient-Generated Subjective Global Assessment (PG-SGA)^(9)^ is a tool for nutritional diagnosis that combines known prognostic indexes (changes in weight and in functional capacity), clinical aspects of food intake, factors that increase the metabolic demand, and physical examination. This evaluation has been indicated as the method of choice for detecting malnutrition among patients with cancer, and is validated specifically for this population^(9,10)^.

Despite concerns about the nutritional status of inpatients, very little attention has been given to acceptance of the hospital diet. Specifically among adult patients with cancer, studies that evaluate food intake plus nutritional status detected by PG-SGA are scarce.

## OBJECTIVE

To verify the acceptance of hospital diets and the nutritional status among patients with cancer admitted in an Oncology/Hematology Unit of a tertiary care hospital.

## METHODS

A cross-sectional study was carried out between July 2011 and February 2012 with men and women aged over 18 years, admitted in the Oncology/Hematology Unit of a tertiary care hospital in Porto Alegre (RS). The unit has a capacity for 30 beds, covered exclusively by the national Unified Healthcare System (SUS).

Simple random sampling was used. Individuals with a minimum length of stay of 3 days (so that the patient would have prior contact with the meals of the hospital diet) and maximum of 6 days (to avoid an acceptance bias related to the monotony of the menus) were consecutively enrolled in the Unit, where the researchers daily obtained the names of the new patients admitted for initial screening. All participants signed the Informed Consent Form and the protocol was approved by the Research Ethics Committee of Hospital Nossa Senhora da Conceição (protocol CEP #10/114). Patients with no condition to answer the questionnaire, who were in NPO (*nil per os*) at the time of the study, receiving foods by means of tube feeding or diets in preparation for exams, inpatients diagnosed with benign hematological diseases or with no confirmed diagnosis of cancer were excluded. All hospitalized individuals were evaluated only once during the study, regardless the number of hospitalizations.

The nutritional risk among adults was detected by the Nutritional Risk Screening (NRS)^(6)^ and among the elderly, by the Mini Nutritional Assessment (MAN)^(7)^. Nutritional status was assessed by objective and subjective methods, and for the anthropometric evaluation, weight in kilograms (kg) and height in meters (m) were obtained directly from the nutritional evaluation in electronic medical records. Body mass index (BMI, kg/m^2^) was calculated and classified as the cutoff points suggested by the World Health Organization (WHO)^(11)^.

The PG-SGA^(9)^ was used as a subjective evaluation method and was applied as follows: the patient completed the first part of the questionnaire, composed by questions as to weight changes, nutritional impact symptoms (nausea, vomiting, lack of appetite, constipation, and diarrhea), changes in food intake and in functional capacity. Illiterate patients had the help of family members for filling in the tool. Trained dietitians completed the second part, which covered aspects of the clinical history of the disease and physical examination. At the end, the evaluator classified the patient as: A) well-nourished, B) moderately malnourished or suspected malnutrition, and C) severely malnourished.

The percentage of diet acceptance was determined by the rest-ingestion index, established as the difference between the quantities of food rejected and the quantity of the meal offered^(12)^. Thus, the rest of the food not ingested by the patients at the noon meal was quantified in grams (g) by weighing the food residue that remained on the thermal plate using Toledo^®^ electronic scales with a capacity of up to three kilograms and specificity of one gram. The leftovers were stored in plastic containers, separated by patient and type of preparation (rice, beans, meat, and accompaniment) for further verification.

Standardization of the measurements was made for proportioning foods on the thermal plate, after a technician in nutrition identified differences among the nutrition employees regarding quantities served. Following repeated verifications of the portions served by the same employees with appropriate tools, the variability among them was reduced and the portions were correctly standardized. The technician in nutrition, who habitually supervised this process, also strictly observed proportions of the foods. These measurements were quantified in grams (g) and were used as a parameter of the meal distributed for the calculation of the rest-ingestion index.

Demographic variables and the reasons for non-acceptance of the meal were verified by means of questionnaire, with questions about taste, appearance, odor, and quantity, time of meal, temperature, and conditions reported by the patient (gastrointestinal disorders and lack of appetite) on the day of the data collection. This questionnaire was made based on an instrument previously used at the hospital^(13)^, adjusted according to the characteristics of this study. Other data of relevance (diagnosis of the disease and treatment given) were obtained from the medical records.

### Statistical analysis

Sample calculation was made by WinPepi software (version 11.18). Considering a significance level of 5%, an estimate of the meal acceptance of 50%^(13)^, and an acceptable difference of up to 10% between well-nourished and malnourished inpatients, the minimal number of participants to be evaluated would be 97 individuals.

Data were organized using Microsoft Office Excel^®^ and the Statistical Package for the Social Science (SPSS) software (version 17.0) was used for statistical analyses. Quantitative variables were described as means and standard deviations or medians, and the categorical variables as percentages. Student's *t* test, Wilcoxon Mann-Whitney test, and Pearson's χ^2^ were used for comparisons; p<0.05 was considered significant.

## RESULTS

One hundred patients were evaluated, with a mean age of 51.6±13.5 years, 59% males and 56% were illiterate/elementary education complete. Malnourished individuals were older and had a higher number of medications prescribed when compared with those who were well-nourished (Table 1).

**Table 1:** Characteristics of participants as per nutritional status

Characteristics		Total sample n = 100	Well-nourished n = 67	Malnourished n = 33	p value
Age (years)		51.6±13.5	49.5±14.5	56±10	0.02*
Gender (%)					0.05**
	Male	59	74.6	25.4	
	Female	41	56.1	43.9	
Schooling (%)					0.5*
	Illiterate/elementary education	56	64.3	35.7	
	High school/further education	44	70.5	29.5	
Length of hospital stay (days)		4.4±1.1	4.4±1.4	4.3±1.1	0.7*
Number of drugs prescribed		5.2±2.8	4.8±2.8	6.1±2.9	0.05**

* Student's *t* test;

** Pearson's χ^2^ test.

Patients were considered malnourished when classified as PG-SGA B or C (33%) and well-nourished (67%) with a PG-SGA A. Mean BMI of the sample was 25.7±6kg/m^2^, and 6.3% of these patients were classified as malnourished according to this criterion (<18.5kg/m^2^). Malnourished inpatients detected by PG-SGA had lower BMI values when compared with the well-nourished individuals (22.4±4.8 *versus* 27.3±6; p<0.0001). There was a nutritional risk among 52% of the patients evaluated. The most prevalent malignancies were lymphomas (22%), digestive tract tumors (21%), head/neck tumors (21%), and leukemia (16%). Treatment most often used was exclusive chemotherapy (51%) or chemotherapy associated with surgery (15%). About 34% of all patients had other diseases, mainly hypertension (22%) or hypertension associated with diabetes mellitus (5%).

**Figure 1:**
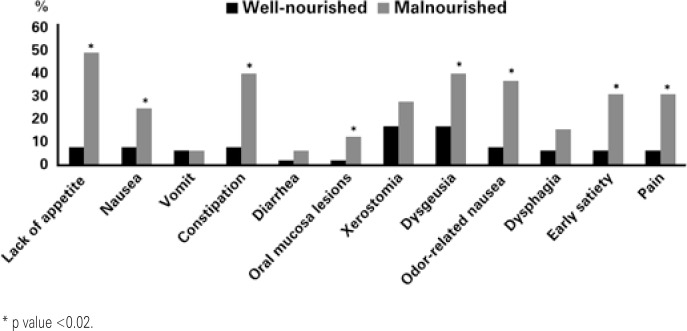
Comparing report of gastrointestinal symptoms among well-nourished and malnourished patients

The main symptoms reported in PG-SGA were lack of appetite (21%), xerostomia (20%), constipation (18%), dysgeusia (17%), odor-related nausea (17%), and early feeling of satiety (14%). Malnourished patients had significantly more complaints of lack of appetite, nausea, constipation, oral mucosal lesions, dysgeusia, odor-related nausea, and pain (p<0.05). Figure 1 shows the comparison between gastrointestinal symptoms reported among the well-nourished and malnourished patients. About the diets prescribed, 70% of the patients received food with unchanged consistency, and with no restriction of any nutrient. Diets with sodium restrictions were prescribed for 22% of the individuals and 12% of the participants received diets with changes in consistency and/or restrictions of some nutrient.

Rest-ingestion index of the noon meal was approximately 37%, and was significantly higher among malnourished patients (p=0.04). About the preparations, rice showed the highest percentage of rejection. Compared with the well-nourished patients, those who were malnourished showed worse acceptance of rice and meat (p<0.05). Table 2 shows the percentage of rest-ingestion index of the meal and of each preparation provided at the noon meal, as per nutritional status.

**Table 2:** Percentage of rest-ingestion index of meals provided as per nutritional status

Food	Total sample n = 100	Well-nourished n = 67	Malnourished n = 33	p value*
Rice	45.5	45.0	61.6	0.006
Beans	33.6	48.0	55.6	0.2
Meat	26.6	46.8	58.0	0.04
Accompaniment	42.1	49.0	51.0	0.7
Total meal	36.9	46.4	58.8	0.04

* Wilcoxon Mann Whitney test.

**Figure 2:**
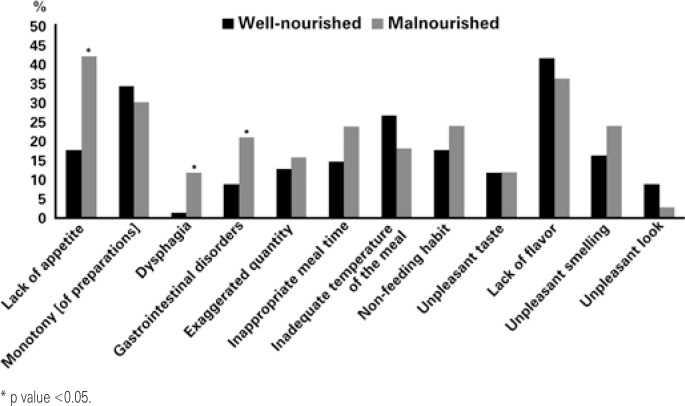
Reasons reported for non-acceptance of meals among well-nourished and malnourished patients

The most frequent reasons reported for non-acceptance of the diet were lack of flavor (40%), monotony of preparations (33%), exaggerated quantity (29%), lack of appetite (26%), and inadequate temperature of the meal (24%). Malnourished patients had significantly more complaints related to lack of appetite, dysphagia, and exaggerated quantity. Figure 2 shows the reasons reported for non-acceptance of the diet, as per the nutritional status.

## DISCUSSION

The present study identified a high rest-ingestion index among patients with cancer, especially among those diagnosed with malnutrition. It was also observed a significant number of gastrointestinal symptoms and complaints reported by malnourished patients. Several authors have already demonstrated that Subjective Global Assessment and its versions seems to detect malnourished with greater precision among hospitalized patients when compared to other methods of nutritional assessment^(2,14–16)^. In the present study, patients considered malnourished by PG-SGA would be classified as “well-nourished” (in good general conditions) according to the BMI diagnosis criterion. Several tools for the evaluation of the nutritional status based on objective methods (anthropometrics, serum protein dosing, cell immunity evaluation, body composition assessment) and on subjective methods were proposed to improve the diagnosis. However, the use of some objective standardized tools among specific ages and diseases (without adequate cut-off points) may difficult the assessment of the real diagnosis of malnutrition. BMI, for example, has as limitation the low level of accuracy in discriminating fat mass and lean mass. The SGA, a method that considers functional and body composition alterations, becomes very useful in clinical practice and correlates positively with objective parameters of nutritional assessment^(17)^.

Another aspect to be considered in PG-SGA is the possibility of evaluating the symptoms of nutritional impact that may affect the patient with cancer^(1,9,10)^. It was further observed that malnourished patients had more complaints regarding these symptoms; the main complaint, lack of appetite, is related to the low acceptance rate of the hospital diet. In some studies, this is one of the main reasons given for reduction in feeding at hospitals^(14,18,19)^.

Rest-intake index found in this study was high and corroborated with results detected by other researchers^(3,20)^, in which a rest-intake index higher than 20% among ill populations may indicate inadequacy in planning and/or executing the menu and is also associated with increased hospital morbidity^(21)^ and mortality^(18)^.

Malnourished patients evaluated in this study showed a higher rest-ingestion index when compared with those who were well-nourished. Study detected that 55% of the malnourished and 35% of the well-nourished individuals consumed less than half of the food offered in hospitals in Australia and New Zealand. When analyzed separately, patients with cancer had 80% greater chances of intake less than 50% of the meal^(15)^. A possible explanation for the fact that even well-nourished patients reduce their food intake during long periods of hospitalization could be associated with the monotony of the menu in the hospital environment^(4,5)^ (with a little variety in diets and meal options, especially in public hospitals) and the effects of drug therapy throughout treatment, with a consequent increase in signs and symptoms, such as lack of appetite and nausea^(1,22,23)^.

Malnourished patients had a worst acceptance of rice and meat in this study. The refusal of certain foods may be associated with the symptoms caused by antineoplasic therapy^(22,23)^, and patients at nutritional risk frequently report food rejection to certain preparations^(19)^. The reasons given for non-acceptance of the diet offered also corroborated with those already described in other publications^(5,24,25)^. Another aspect is that malnourished patients considered exaggerated the quantity of foods served and some authors suggest that individuals consider the meals more appealing at hospitals when they are served in small portions^(19)^.

The present study evaluated the acceptance of the meals by direct weighing of the leftovers of each preparation, offering more precise data about food intake and the types of food most tolerated by the patients. One of the limitations of this study is the fact that foods served on the thermal plate were not weighed at the time of measuring out the portions of the meal. Another consideration is about the cross-sectional design, which does not directly characterize the risk of malnutrition due to the percentage of the meal not ingested.

## CONCLUSION

Patients with cancer had a high rest-ingestion index, and a highest percentage among malnourished inpatients according to PG-SGA was detected when compared to well-nourished ones. The subjective evaluation seems to be a better tool than BMI for diagnosing malnutrition in this population and symptoms of nutritional impact were reported by a large part of the inpatients, especially among malnourished.

The results obtained in this study reinforce the importance of evaluation and nutritional follow-up in clinical practice, as well as monitoring of food intake among patients with cancer, who are individuals vulnerable to malnutrition. In this regard, diet techniques and hospital gastronomy are essential for the preparation of nourishing menus that stimulate the ingestion of food by patients. More studies that evaluate hospital diet acceptance among patients with cancer should be conducted, with the objective of early nutritional intervention in order to avoid a negative evolution of the nutritional status.
